# Renal Medullary Carcinoma Masquerading as Bilateral Breast Carcinoma Category: Case Report

**DOI:** 10.4021/wjon676w

**Published:** 2013-07-15

**Authors:** Madhurima Anne, Daniel Sammartino, Shweta Chaudhary, Tawfiqul Bhuiya, Bhoomi Mehrotra

**Affiliations:** a Division of Hematology-Oncology, Hofstra North Shore - LIJ School of Medicine, Lake Success, NY, USA; b Department of Medicine Hofstra North Shore - LIJ School of Medicine, Lake Success, NY, USA; c Department of Pathology, Hofstra North Shore - LIJ School of Medicine, Lake Success, NY, USA; d Department of Hematology/Oncology, St. Francis Hospital, Roslyn, NY, USA

**Keywords:** Renal cell carcinoma, Renal medullary carcinoma, Breast metastasis, Sickle cell trait

## Abstract

Metastatic disease to the breast accounts for less than 1% of all breast carcinoma. Here we describe an unusual case of a 34-year-old black female with history of sickle cell trait who presented to her gynecologist with bilateral palpable breast masses. Based on initial workup including pathology results from biopsies of both breast masses, she was diagnosed with bilateral breast cancer. However further radiographic imaging revealed a large right kidney mass suspicious for primary renal neoplasm along with lung and bone lesions. This prompted re-review of the initial breast pathology. Sickled erythrocytes were identified and results of an additional immunohistochemical panel revealed positive expression of PAX 8, vimentin, Oct3/4, and loss of INI1, confirming the diagnosis of metastatic renal medullary carcinoma. We discuss the importance of considering renal medullary carcinoma in the differential diagnosis when evaluating young patients with sickle cell hemoglobinopathies who present with aggressive metastatic disease.

## Introduction

The majority of malignant breast disease occurs from a primary breast carcinoma. Although rare for any extramammary malignancy to metastasize to the breast, tumors which may present with breast metastasis include leukemia, lymphoma, melanoma, lung, neuroendocrine, and in children rhabdomyosarcoma. Renal tumors which are known for their unusual pattern of spread only account for about 3% of cases [1-4]. Renal medullary carcinoma is a rare malignancy that predominantly affects patients with sickle cell hemoglobinopathies, mainly sickle cell trait. It has been described as an aggressive cancer, which is nearly universally metastatic at presentation [5]. We report the first described case of renal medullary carcinoma presenting with breast metastases posing a diagnostic challenge.

## Case Report

**Figure 1:**
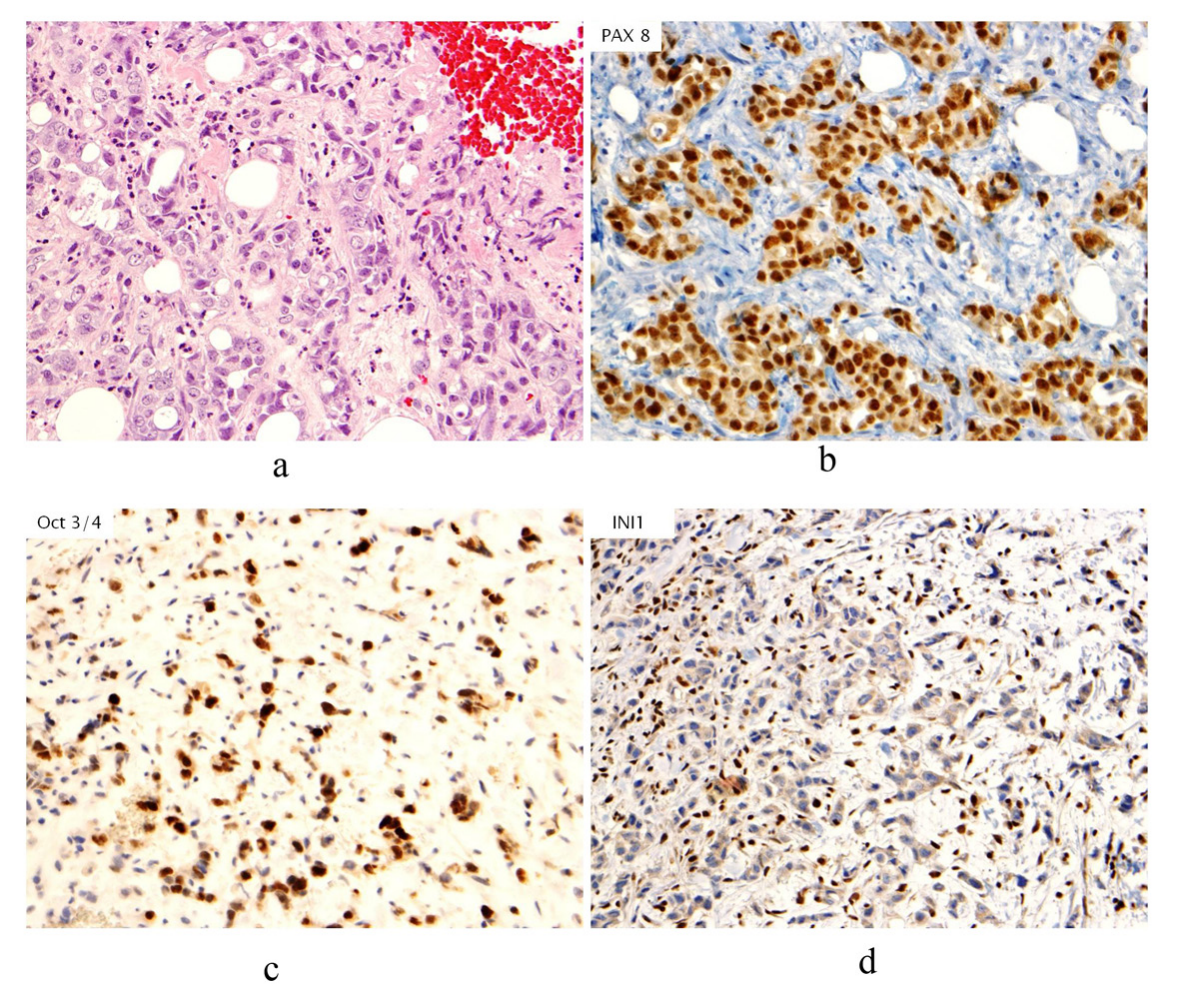
(a). Hematoxylin & Eosin stain (H&E stain) of biopsied breast tissue showing infiltrative, poorly differentiated carcinoma growing in cords and forming occasional tubules. No associated ductal carcinoma in situ component, microcalcifications or lymphovascular involvement by tumor is seen; (b). Immunohistochemical stain of PAX8 revealing nuclear staining in neoplastic cells; (c). Immunohistochemical stain of Oct3/4 revealing nuclear staining in neoplastic cells; (d). Immunohistochemical stain of INI1 showing absence of staining in neoplastic cells.

**Figure 2:**
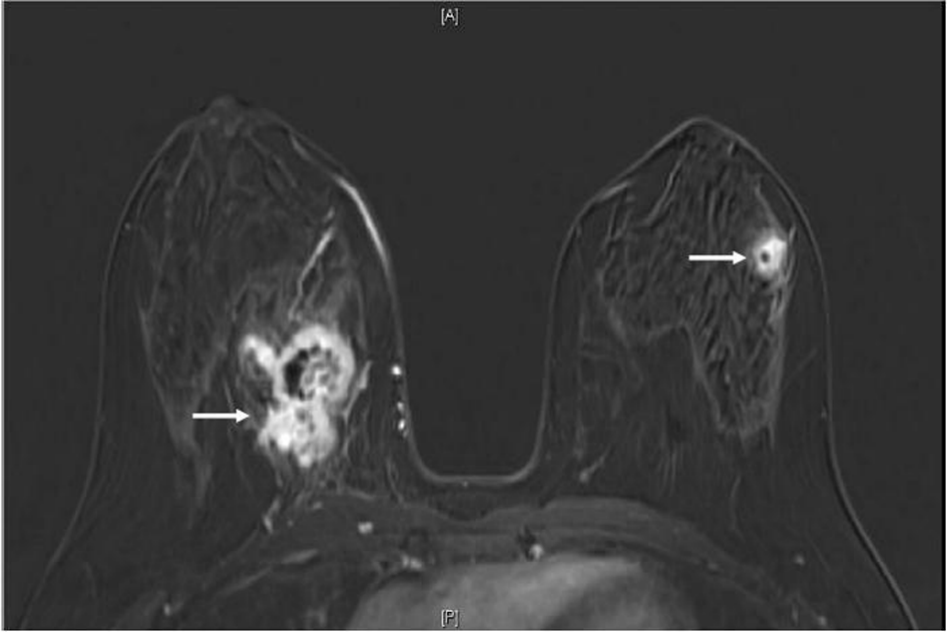
MRI breast (T1, fat saturated, post-contrast subtraction image) showing the largest right breast mass (left arrow) which is heterogeneously enhancing, with central necrosis and the largest left breast mass (right arrow), also heterogeneously enhancing with central necrosis.

A 34-year-old female with past medical history of sickle cell trait presented to her gynecologist with bilateral palpable breast masses. She was referred for bilateral mammograms and sonograms which revealed a 4.2 × 2.2 × 3.2 cm irregular right breast mass and 1.3 × 1.0 × 0.8 cm left breast mass, highly suspicious for malignancy. She underwent ultrasound-guided core biopsies of both lesions which showed infiltrative, poorly differentiated carcinoma (Fig. 1a). Immunohistochemistry performed for estrogen receptor, progesterone receptor and human epidermal growth factor receptor 2 were negative and Ki67 proliferative index was approximately 70%. At this time, the patient was referred to the oncology clinic. The patient underwent bilateral breast magnetic resonance imaging (MRI) which revealed multiple suspicious enhancing masses in all four quadrants of the right breast, a possible right pleural based enhancing mass and multiple suspicious masses in the left breast (Fig. 2). She was sent for Computed Tomography (CT) of the chest with intravenous contrast to evaluate the pleural mass and was discovered to have multiple pulmonary nodules. A CT-guided fine needle aspiration of a 1.9 × 1.2 cm right lower lobe lung nodule showed malignant cells similar to those from the breast biopsies. Subsequent bone scan revealed multifocal osseous disease involving the thoracic, lumbar spine and right ischium, acetabulum and superior ramus. Options for systemic chemotherapy for the treatment of metastatic triple negative breast carcinoma were discussed at this time.

**Figure 3:**
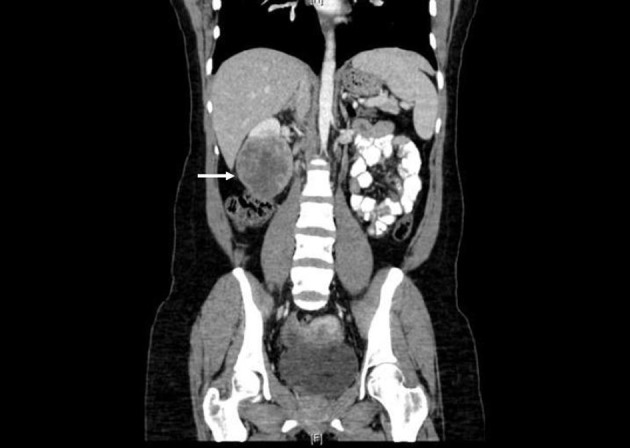
Axial CT (with oral and intravenous contrast enhancement) showing a 6.7 × 6.9 cm heterogeneous exophytic mass with areas of central low attenuation arising from the lower pole of the right kidney, with areas of central necrosis.

**Figure 4:**
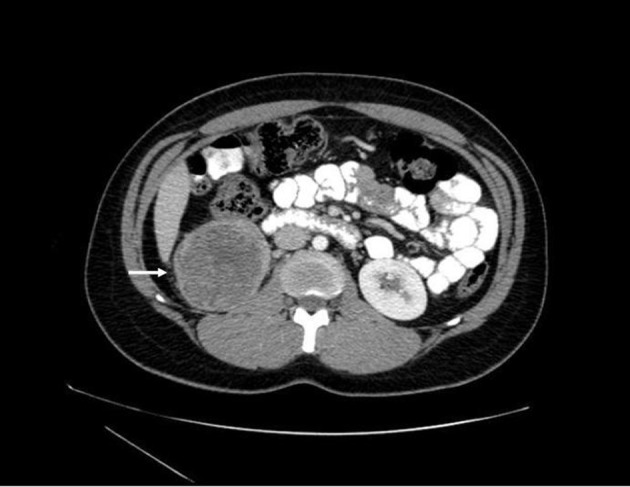
Coronal CT (with oral and intravenous contrast enhancement) showing large heterogeneously enhancing mass replacing lower pole of the right kidney.

Prior to treatment, she underwent contrast enhanced staging CT of the abdomen and pelvis which revealed a 6.7 × 6.9 cm mass arising from the lower pole of the right kidney (Fig. 3, 4). Given the patient’s young age, history of sickle cell trait, radiologic findings of a large renal mass, multiple lung and breast nodules as well as the histological picture, the diagnosis of metastatic renal medullary carcinoma was considered. At this time, the original breast core biopsies were re-reviewed. Histological findings of prominent neutrophilic infiltrate closely associated with the tumor and sickled erythrocytes were identified. An additional immunohistochemical panel of PAX 8, vimentin, gross cystic disease fluid protein (GCDFP), Oct 3/4 and INI1 (BAF47) was performed. Positive expression of PAX 8 (nuclear) (Fig. 1b), vimentin (cytoplasmic and membranous), Oct3/4 (nuclear) (Fig. 1c) and loss of INI1 expression (Fig. 1d) in tumor cells confirmed the diagnosis of renal medullary carcinoma metastatic to breast [6-8].

The new diagnosis of metastatic renal medullary carcinoma was discussed with the patient. Retrospective review of outpatient medical records did reveal microscopic hematuria dating eight months prior to her diagnosis. At this time her poor prognosis was discussed and she opted for systemic chemotherapy. She was started on carboplatin and gemcitabine and achieved a partial response after two cycles with regression of the kidney tumor and lung lesion. After four cycles she developed progressive osseous metastatic disease with stable systemic disease and required radiation to her right hip. She then developed spinal cord compression requiring radiation as well as systemic progression of disease. At this point she was switched to weekly paclitaxel and received four cycles before her performance status declined and she died of disease progression twelve months after initial diagnosis.

## Discussion

Renal medullary carcinoma is a rare malignancy, first described in 1995 by Davis et al, as a malignancy that predominantly affects young black patients with sickle cell trait with a male predominance [9]. The hypoxic, acidotic environment of the renal medulla is thought to promote the polymerization of hemoglobin S in patients with sickle cell trait causing sickling in this area of the kidney. This is thought to stimulate the proliferation of epithelial cells in the region which can lead to the development of malignant growth [10, 11]. It has also been postulated that the expression of transcription factor, hypoxia inducing factor (HIF) is increased in the hypoxic environment. In normal cells, during states of cellular hypoxia, HIF induces TP53 leading to cell death. However, in tumors lacking p53, HIF upregulates vascular endothelial growth factor (VEGF), leading to angiogenesis thus further stimulating tumor growth and progression in a proliferating environment [12, 13]. This theory has been supported by immunohistochemical analyses demonstrating increased expression of HIF, VEGF, and TP53 protein in these tumors [13].

The most common presentation of renal medullary carcinoma includes gross hematuria, abdominal or flank pain, weight loss, fever, as well as an abdominal mass [13, 14]. Patients generally present with advanced metastatic disease that is poorly responsive to chemotherapy. The most commonly reported sites of metastases include regional lymph nodes, lung, adrenal gland and liver [9, 13, 15]. Imaging can be helpful to distinguish renal medullary carcinoma from renal cell carcinomas. Unique radiographic features which have been observed in patients with renal medullary carcinoma include a strong right sided predominance, centrally located tumor, hypovascularity, heterogeneous contrast enhancement due to areas of necrosis, retroperitoneal adenopathy, infiltrative growth causing caliectasis, and intra-tumoral hemorrhage [13, 15-17]. Although CT or MRI findings can be suggestive, a fine needle aspiration biopsy should be performed for histologic diagnosis.

The morphology of renal medullary cancer cells on light microscopy can be quite variable. Frequently observed characteristics include large vesicular nuclei with prominent nucleoli, eosinophilic cytoplasm, a reticular pattern of growth, a desmoplastic stroma with inflammatory infiltrate, areas of hemorrhage and infiltrative margins. Sickled erythrocytes are also frequently observed [5, 10, 12, 13]. In a series by Swartz et al, immunohistochemical studies demonstrated expression of low molecular weight cytokeratin (CAM 5.2), epithelial membrane antigen, vimentin, HIF and VEGF, with variable expression of cytokeratins 7 and 20 [13]. In addition, loss of INI 1 and strong nuclear expression of OCT3/4 has been described in these tumors [6, 8].

Prognosis is poor for patients diagnosed with renal medullary carcinoma. There is no standard treatment of choice for this aggressive disease. In a case series by Simpson et al, data for 28 patients treated with different regimens were reviewed and a mean survival was calculated to be 32 weeks (range of 2 to 62 weeks). A 12 month survival with a commonly used regimen, methotrexate, vinblastine, doxorubicin and cisplatin (MVAC) has been reported; when given in high dose with granulocyte colony-stimulating factor support, a 16 month survival has been described in one patient [12]. Treatment with a combination of carboplatin, paclitaxel and gemcitabine has been reported with a mean survival of 11 months. Other agents which have been used in this disease include vinblastine, ifosfamide, etoposide, interferon, interleukin-2 and combination of topotecan, doxorubin and filgrastim [18-20]. One case of a patient treated with thalidomide has been reported with a 52 week survival. Most recently, a carboplatin and paclitaxel combination, given to two patients at three week intervals has been described with survival times of 15 and 11 months [12]. Despite some initial activity of these regimens in renal medullary carcinoma, in all patients there was eventual rapid progression of disease and demise.

### Conclusion

While the majority of breast masses are secondary to a primary breast cancer, the importance of complete pathological and radiologic review prior to surgical or medical management of breast cancer can be illustrated in this case. In a young patient with sickle cell trait who presents with hematuria, renal medullary carcinoma should be considered with prompt workup for accurate diagnosis and treatment. While there have not been any large advances in treatment with standard chemotherapeutics, future developments for treatment of this malignancy may include targeted anti-angiogenesis therapies given increased expression of VEGF in these tumors.
